# Gene Prediction in Metagenomic Fragments with Deep Learning

**DOI:** 10.1155/2017/4740354

**Published:** 2017-11-08

**Authors:** Shao-Wu Zhang, Xiang-Yang Jin, Teng Zhang

**Affiliations:** Key Laboratory of Information Fusion Technology of Ministry of Education, School of Automation, Northwestern Polytechnical University, Xi'an 710072, China

## Abstract

Next generation sequencing technologies used in metagenomics yield numerous sequencing fragments which come from thousands of different species. Accurately identifying genes from metagenomics fragments is one of the most fundamental issues in metagenomics. In this article, by fusing multifeatures (i.e., monocodon usage, monoamino acid usage, ORF length coverage, and Z-curve features) and using deep stacking networks learning model, we present a novel method (called Meta-MFDL) to predict the metagenomic genes. The results with 10 CV and independent tests show that Meta-MFDL is a powerful tool for identifying genes from metagenomic fragments.

## 1. Introduction

Metagenomics bypasses the needs for isolation and lab cultivation of individual species, and the uncultured microbes are sampled directly from their environment [1–3]. High-throughput sequencing technology used in metagenomics can yield millions of short DNA/RNA fragments (or reads) in a single run for the environmental samples and helps us uncover the microbial diversity and better understand how these unknown microbes live and coexist with unprecedented resolution. However, it is in many cases impossible to reliably assemble these short reads into longer contigs because the metagenomic sequencing reads come from thousands of highly uneven different species, and it cannot provide a high sequencing coverage of single species. Several de novo metagenomic short read assemblers such as Meta-IDBA [4], IDBA-UD [5], Ray Meta [6], MetaVelvet-SL [7], Omega [8], and metaSPAdes [9] have shown promising results and effectiveness in assembling metagenomic short reads, but they do not work well in cases where there are more species presenting in the environmental samples [10]. Thus, one way to analyze the metagenomics data is to bypass assembly and go directly finding the genes from these short reads. Gene recognition is a necessary step to fully understand the functions, activities, and roles of genes in cellular processes.

Accurate gene prediction in metagenomes is more complicated than in isolated genomes [11–13]. One reason is that most fragments (reads) from the high-throughput sequencing technologies are very short [11, 14]. Lots of genes are incomplete with one or two ends exceed the fragments, and a single fragment usually contains only one or two genes. Another reason is that the source genomes of the fragments are always unknown or totally new, which brings challenge on statistical model construction and feature selection [11].

Until now, several methods have been developed to predict genes from metagenomics DNA fragments. These prediction approaches can be categorized into homology-based methods, model-based methods, and machine learning-based methods. The homology-based methods such as CRITICA [15] and Orpheus [16] often used the BLAST package to compare the input fragments against known protein databases for analyzing the short sequences. However, these methods just are used to find the genes with previously known homologous proteins and cannot predict novel genes. The model-based methods, such as MetaGeneAnnotator [17], MetaGene [18], MetaGeneMark [19], FragGeneScan [14], and Glimmer-MG [13], used either the higher-order Markov chain models or the hidden Markov chain models to identify genes in metagenomics. However, the main limitation of such Markov chain models is that thousands of parameters are needed in practical use. The machine learning-based methods such as Orphelia [20, 21], MGC [22], and MetaGUN [11] often formulated the metagenomic fragments with an effective mathematical expression that can truly reflect their intrinsic correlation with the target to be predicted and then designed a powerful classifier with machine learning to operate the gene prediction. Orphelia [20, 21] integrates monocodon and dicodon usage, sequence patterns around translation initiation sites (TISs), open reading frame (ORF) length, and GC-content to represent the ORF fragments, then inputting into an artificial neural network to estimate the probability that a given ORF encodes a protein. As an improvement over the Orphelia algorithm, MGC [22] learns separate models for several predefined GC ranges as opposed to the single model used in Orphelia and applies the appropriate model to each fragment based on its GC-content. That is, MGC uses a two-stage machine learning approach to predict genes by firstly computing six features (i.e., monocodon score, dicodon score, monoamino acid score, diamino acid score, TIS coverage, and TIS probability) from ORF with the corresponding linear discriminant, then estimating the gene probability of a given ORF with the neural network model from the corresponding GC range, which takes nine features of the monocodon score, dicodon score, monoamino acid score, diamino acid score, TIS coverage and TIS probability, length complete, length incomplete, and GC-content as input. MetaGUN [11] implements a three-stage strategy to predict genes by firstly classifying input sequence into different phylogenetic groups, then identifying genes for each group independently with support vector machine (SVM) classifier that integrate entropy density profile of codon usage, translation initiation site scores, and ORF length as the input features, and finally adjusting TISs by employing a modified version of MetaTISA. Although the existing metagenomics gene predictors or methods can effectively identify genes, these predictors employ the shallow architectures such as hidden Markov models (HMMs), SVMs, and multilayer perceptron (MLP) with a single hidden layer. A common property of these shallow learning models is the simple architecture that consists of only one layer responsible for transforming the raw input features into a problem-specific feature space [23], which has been shown effective in solving many simple or well-constrained problems, but its limited modelling and representational power can cause difficulties when dealing with more complicated real-world applications [24].

To further enhance metagenomic gene prediction accuracy, in this study, we developed a new powerful predictor (named as Meta-MFDL) by fusing multiple features of the ORF length coverage, monocodon usage, monoamino acid usage, and Z-curve features and employing the deep learning classification algorithm. Deep learning is a new area of machine learning research, which attempt to model high-level abstractions in data by using model architectures composed of multiple nonlinear transformations [24, 25]. Now, some deep learning architectures such as convolutional deep neural networks (CNNs), deep belief networks (DBNs), deep neural networks (DNNs), and deep stacking networks (DSNs) have been applied to improve the performance in image and speech recognition [26, 27], in natural language processing [28, 29], and most recently in computational biology [30, 31], such as protein structure and subcellular localization prediction [32–35], gene expression regulation regarding splice junctions or RNA binding proteins [36–42], lncRNA prediction [24], and metagenomic classification [43]. In comparison with the existing predictors, Meta-MFDL showed better performance on the training datasets (i.e., Set700 and Set120) derived from 120 complete genomes in 10-fold cross validation (10 CV) and independent testing datasets (i.e., ^Tes^Data700 and ^Tes^Data120) derived from 13 genomes.

## 2. Materials and Methods

### 2.1. Datasets

Genomic sequences and annotation information of 120 complete genomes (i.e., 109 bacteria and 11 archaea) and 13 complete genomes (i.e., 10 bacteria and 3 archaea) are obtained from NCBI RefSeq database for separately constructing the training and testing datasets. The genomes of the 13 species used in previous methods [20–22] are not included in the training dataset. All genomes information in the training and testing datasets is listed in Tables S1 and S2 in Supplementary Material available online at https://doi.org/10.1155/2017/4740354. In order to simulate DNA sequences from different sequencing technologies, the genome sequences in training dataset are randomly split into 700 bp and 120 bp fragments with 1-fold genome coverage for each genome, and the Metasim [44] is used to generate 700 bp and 120 bp fragments with the 3-fold genome coverage for each genome in the testing dataset. Now that the purpose of metagenomics gene prediction is to discriminate the coding ORFs from the noncoding ORFs; thus we extracted all ORFs from these fragments and divided these ORFs into coding and noncoding ORFs based on the annotation of the genome. ORFs obtained can be categorized into complete and incomplete ORFs. The complete ORFs have both the start codon and the stop codon. The incomplete ORFs lack the upstream end, the downstream end, or both ends in which case the ORF holds the whole fragments without starting codons or stopping codons [21, 22]. Only ORFs with a minimal length of 60 bp are kept in both training and testing datasets, for the ORFs less than 60 bp is too short to provide useful information [11, 17]. After strictly following the above procedures, we finally obtained two training datasets of ^Tra^Data700 and ^Tra^Data120 and two testing datasets of ^Tes^Data700 and ^Tes^Data120. ^Tra^Data700 dataset consists of 1,654,069 coding ORF fragments and 2,267,896 noncoding ORF fragments. ^Tes^Data700 dataset consists of 303,664 coding ORF fragments and 423,078 noncoding ORF fragments. ^Tra^Data120 dataset consists of 5,204,861 coding ORF fragments and 5,812,025 noncoding ORF fragments. ^Tes^Data120 dataset consists of 548,357 coding ORF fragments and 612,049 noncoding ORF fragments. The ^Tra^Data700, ^Tes^Data700, ^Tra^Data120, and ^Tes^Data120 datasets can be downloaded from http://180.208.58.19/Meta-MFDL/.

In general, establishing a highly useful predictor involves the following five steps [24, 45, 46]: (1) constructing a valid benchmark dataset to train and test the predictor; (2) using effective mathematical expression to convert the nucleotide (or protein) alphabetic sequences into feature vectors that truly reflect their intrinsic correlation with the attribute to be predicted; (3) developing/choosing a powerful algorithm to operate the prediction; (4) properly selecting the cross-validation tests to objectively evaluate the performance of the predictor; and (5) establishing a software tool. The Meta-MFDL predictor can be divided into three steps: feature extraction, feature fusion, and pattern classification. For feature extraction which is one of the most critical steps in designing a classifier, the query ORF fragments are converted into a series of vectors with the ORF coverage (ORFC), monocodon usage (MCU), monoamino acid usage (MAU), and Z-curve descriptors. For feature fusion, the four kinds of features of ORFC, MCU, MAU, and Z-curve are integrated to represent the candidate ORF fragments. For pattern classification, the vectors are classified by using the deep stacking networks, one of the deep learning architectures.

### 2.2. Feature Descriptors

#### 2.2.1. ORF Coverage Descriptor

Previous study shows that the length of coding ORFs is considerably longer than that of noncoding ORFs [47]. Thus, we can use the following feature vector *X*_ORFC_ to represent metagenomic fragment by computing the length proportion of the ORF to the fragment:

(1) $$\begin{array}{c} X_{ORFC}=\frac{l}{L}, \end{array}$$

where *l* is the ORF length and *L* is the length of fragment.

#### 2.2.2. Monocodon Usage Descriptor

Codon usage is a useful feature in discriminating coding and noncoding ORFs [21]. Here, we use the following vector *X*_MC_ to represent one fragment by counting the monocodon frequency of ORF (including the complete ORF fragment and incomplete ORF fragment):

(2) XMC=f1c,…,fic,…,f64cfic=niN,

where *f*_*i*_^*c*^ is the frequency of *i*th monocodon (e.g., AAA, AAC,…, UUG, UUU, and three stop codons of UGA, UAA, and UAG) occurring in ORF, *n*_*i*_ is the number of *i*th codon in ORF, and *N* is the number of all codons in ORF.

#### 2.2.3. Monoamino Acid Usage Descriptor

The monoamino acid usage is the 21 amino acid (20 amino acids plus one “STOP” codon) frequencies occurring in the DNA sequences, some of which are linearly related to the GC-content of the genome [48] and are important in discriminating coding and noncoding DNA sequences [22]. Here, we use the following vector *X*_MA_ to represent one fragment by counting the monoamino acid frequency of ORF (including the complete ORF fragment and incomplete ORF fragment).

(3) $$\begin{array}{c} X_{MA}=\left[\;f_{1}^{aa},\ldots ,f_{j}^{aa},\ldots ,f_{21}^{aa}\;\right], \end{array}$$

where *f*_*j*_^aa^ is the frequency of *j*th amino acid (e.g., A, C, D, E, F, G, H, I, K, L, M, N, P, Q, R, S, T, V, W, Y, and one “STOP” codon) occurring in ORF.

#### 2.2.4. Z-Curve Parameter Descriptor

Z-curve parameter features are calculated for the frequencies of frame-dependent *k*-mers by using the Z-transform of DNA sequences [49] and have been successfully applied to gene prediction [50, 51]. Let *a*_1_, *c*_1_, *g*_1_, *t*_1_; *a*_2_, *c*_2_, *g*_2_, *t*_2_; *a*_3_, *c*_3_, *g*_3_, and *t*_3_ represent the frequencies of bases A, C, G, and T occurring at positions 1, 4, 7, 10,…; 2, 5, 8, 11,…; 3, 6, 9, 12,… and in an ORF fragment, respectively. The* p*_12_(AA),* p*_12_(AC),…,* p*_12_(TT);* p*_23_(AA),* p*_23_(AC),…, and* p*_23_(TT) denote the frequencies of the 16 dinucleotides AA, AC,…, and TT occurring at the codon positions 1-2 and 2-3 of an ORF fragment, respectively. By using the Z-transform of DNA sequence [50], we can use the following vectors *X*_ZCPS_, *X*_ZCPD_ to represent the ORF fragment by counting the frequencies of codon-position-dependent single nucleotides and the frequencies of phase-specific dinucleotides, respectively.

(4) XZCPS=x1,y1,z1,x2,y2,z2,x3,y3,z3xi=ai+gi−ci+tiyi=ai+ci−gi+tizi=ai+ti−gi+cixi,yi,zi∈−1,1,  i=1,2,3XZCPD=x12A,y12A,z12A,…,x12T,y12T,z12T,x23A,y23A,z23A,…,x23T,y23T,z23TxkM=pkMA+pkMG−pkMC+pkMTykM=pkMA+pkMC−pkMG+pkMTzkM=pkMA+pkMT−pkMC+pkMGM=A,C,G,T;  k=12,23.

### 2.3. Feature Fusion

Feature fusion can derive the most discriminatory information from original multifeature sets and eliminate the redundant information from the correlation between distinct feature sets, which benefits the final decision. Here, four kinds of feature set of ORF coverage, monocodon usage, monoamino acid usage, and Z-curve parameters are concatenated into one set of feature vectors to represent the ORF fragments, which can be formulized as follows:

(5) $$\begin{array}{c} X=\left[\;X_{ORFC},X_{MC},X_{MA},X_{ZCPS},X_{ZCPD}\;\right]. \end{array}$$

### 2.4. Deep Stacking Network

As one of the deep neural networks, deep stacking network (DSN) has been successfully applied in speech classification [52], information retrieval [53], and lncRNA identification [24]. A DSN is stacked by a series of modules with the same or similar structures. Each DSN module takes a simplified form of the shallow multilayer perceptron, consisting of a linear input layer, a nonlinear hidden layer and a linear output layer. “Stacking” is accomplished by concatenating the output of all previous modules with the raw input vector to form the new “input” vector as the input of the next module. The DSN weight parameters *W* (input weight matrices) and *U* (output network weight matrices) in each module are learned efficiently from the training data by using the basic learning algorithm and the fine tuning algorithm.

#### 2.4.1. Basic Learning Algorithm

Let **X** = [**x**_1_,…, **x**_**i**_,…, **x**_**N**_] represent the training vectors, in which each vector is denoted by **x**_*i*_ = [*x*_1*i*_,…,*x*_*ui*_,…,*x*_*Di*_]^*T*^; **T** = [**t**_1_,…, **t**_*i*_,…, **t**_*N*_] represents the target vectors, in which each vector is denoted by **t**_*i*_ = [*t*_1*i*_,…,*t*_*vi*_,…,*t*_*Ci*_]^*T*^, where *N* is total number of training samples, *D* is the dimension of the input vector, and *C* is dimension of the output vector. Let *L* denote the number of hidden units. Then, the output of a DSN module is **y**_**i**_ = **U**^**T**^**h**_**i**_, where **h**_**i**_ = *σ*(**W**^**T**^**x**_**i**_) is the *i*th hidden layer output, **U** is a *L* × *C* weight matrix at the upper layer, **W** is a *D* × *L* weight matrix at the lower layer, and *σ*(·) is the sigmoid function. For a given **W**, the parameter **U** is learned to minimize the average of the total square error.

(6) $$\begin{array}{c} E={\left\|\;Y-T\;\right\|}^{2}=Tr\left[\;\left(\;Y-T\;\right){\left(\;Y-T\;\right)}^{T}\;\right], \end{array}$$

where **Y** = [**y**_1_,…, **y**_**i**_,…, **y**_**N**_]. If the lower layer weight matrix **W** is fixed, the hidden layer output values  **H** can also been calculated. Consequently, the upper-layer weight matrix **U** in each module can be determined by setting the gradient ∂*E*/∂**U** = 2**H**(**U**^*T*^**H** − **T**)^*T*^ to zero, then leading to the closed-form solution.

(7) $$\begin{array}{c} U={\left(\;HH^{T}\;\right)}^{-1}HT^{T}. \end{array}$$

In general, there are two ways to set **W**: firstly, by using various distributions to generate the random numbers to set **W**; secondly, by applying contrastive divergence to separately train the restricted Boltzmann machines (RBM), then using the trained RBM weights to set **W**. In this paper, we used the trained RBM weights to set **W** for the bottom module.

#### 2.4.2. Module-Bound Fine Tuning

The weight matrices **W** of DSN in each module can be further learned using the batch-mode gradient descent [54]. That is,

(8) ∂E∂W=∂ TrUTH−TUTH−TT∂W=2XHT∘1−HT∘H†HHTTH†−TTTH†Wj+1=Wj+η×∂Ej∂Wj,

where **H**^†^ = **H**^*T*^(**HH**^*T*^)^−1^ is pseudoinverse of **H**, the symbol ∘ represents the element-wise matrix multiplication, and *η* is the learning rate of updating the weight matrices **W**.

### 2.5. The Performance Measures of the Prediction System

The performance measures of the sensitivity or true positive rate (TPR), precision or positive predictive value (PPV), accuracy (ACC), and* F*1 score were used to evaluate the performance of the prediction system. They are defined as follows:

(9) TPR=TPTP+FNPPV=TPTP+FPF1=2×PPV×TPRPPV+TPRACC=TP+TNTP+TN+FP+FN,

where TP is the number of correctly predicted coding genes and FP and FN are the number of incorrectly predicted coding genes and noncoding gene, respectively.* F*1 score is the harmonic mean of precision and sensitivity, which provides a composite of precision and sensitivity.

## 3. Results and Discussion

### 3.1. Performance of Meta-MFDL

In statistical prediction, the following three cross-validation methods are often used to examine a predictor for its effectiveness in practical application: independent dataset test, *K*-fold (e.g., 5-fold, 10-fold) crossover or subsampling test, and jackknife test. Of the three test methods, the jackknife test is deemed the least arbitrary that can always yield a unique result for a given benchmark dataset [45]. However, for large scale database, the jackknife test needs to spend lots of time to generate the prediction results. To reduce the computational time and evaluate the generalization performance of a predictor, in this study, we adopted the 10-fold cross-validation (10 CV) test and independent dataset test as done by most investigators [24, 46, 55–59].

Generally, more ORF fragments are used to train the deep learning model, higher gene prediction accuracy can be obtained, but it will spend more computational time. By making a trade-off between the prediction accuracy and computational time, we randomly select 50,000 coding ORF fragments and 50,000 noncoding ORF fragments from ^Tra^Data700 and ^Tra^Data120, respectively, to form Set700 and Set120 datasets to train the Meta-MFDL predictor. To demonstrate the superiority of Meta-MFDL, in 10 CV test, we compared it with other two state-of-the-art predictors, Orphelia [21, 22] and FragGeneScan [14]. Orphelia [21, 22] firstly extracts the features of monocodon usage, dicodon usage, and translation initiation sites by building linear discriminants and then combines these features with ORF length and GC-content by using an artificial neural network to estimate the probability of an ORF encoding a protein. FragGeneScan [14] combines codon usage, sequence patterns for start/stop codons, and sequencing error models in a hidden Markov model to improve the prediction of protein-coding region in short reads. In order to show the outstanding performance of deep learning algorithm, we also design a Meta-MFSVM predictor. Meta-MFSVM uses the same features as Meta-MFDL, inputting the support vector machine (SVM) to distinguish between coding and noncoding ORF fragments. The results of four predictors on the same training datasets (i.e., Set700 and Set120) in 10 CV test are shown on Table 1. It can be seen that, for 700 bp dataset, the TPR of Meta-MFDL is 91.47%, which is 2.86, 1.88, and 1.09% higher than that of Orphelia, Meta-MFSVM, and FragGeneScan, respectively; the PPV of Meta-MFDL is 93.26%, which is 4.21, 3.03, and 1.37% higher than that of Orphelia, Meta-MFSVM, and FragGeneScan, respectively;* F*1 score of Meta-MFDL is 0.923, which is 0.035, 0.015, and 0.005 higher than that of Orphelia, Meta-MFSVM, and FragGeneScan, respectively; most deviations of Meta-MFDL are also lower than that of Orphelia, Meta-MFSVM, and FragGeneScan. For 120 bp dataset, the TPR of Meta-MFDL is 89.28%, which is 6.09, 4.58, and 2.72% higher than that of Orphelia, Meta-MFSVM, and FragGeneScan, respectively; the PPV of Meta-MFDL is 90.58%, which is 8.14, 5.51, and 3.8% higher than that of Orphelia, Meta-MFSVM, and FragGeneScan, respectively;* F*1 score of Meta-MFDL is 0.899, which is 0.052, 0.05, and 0.031 higher than that of Orphelia, Meta-MFSVM, and FragGeneScan, respectively; all deviations of Meta-MFDL are lower than that of Orphelia, Meta-MFSVM, and FragGeneScan. The results of 700 bp and 120 bp datasets show that Meta-MFDL can effectively recognize the short coding fragments in metagenomics than Orphelia and FragGeneScan predictors.

In addition, the comparing results of Meta-MFDL and Meta-MFSVM indicate that the classifying ability of deep learning is superior to SVM. These results show that the Meta-MFDL predictor has the powerful performance for distinguishing coding and noncoding ORF fragments.

In order to further evaluate the generalized performance of Meta-MFDL predictor, we also implemented Meta-MFDL on ^Tes^Data700 and ^Tes^Data120 testing datasets derived from 13 species (i.e., 10 bacteria and 3 archaea) and compared with Orphelia [21, 22], FragGeneScan [14], MGC [23], MetaGUN [11], and Meta-MFSVM predictors. The overall accuracies of Meta-MFDL, Meta-MFSVM, Orphelia, FragGeneScan, MGC, and MetaGUN on the two independent testing datasets (i.e., ^Tes^Data700 and ^Tes^Data120) are shown in Tables 2 and 3, respectively. The TPR, PPV, and* F*1 score of these six methods are listed in Tables S3 and S4 available in Supplementary Material. It can be seen that both Meta-MFDL and MetaGUN achieve better performance than Orphelia, Meta-MFSVM, MGC, and FragGeneScan on most species. The performance of Meta-MFDL is slightly better than that of MetaGUN; for example, for the ^Tes^Data700 independent testing dataset, the average accuracy of Meta-MFDL is 93.31%, which is 0.33% higher than that of MetaGUN; the average* F*1 score is 0.9184, which is 0.0029 higher than that of MetaGUN. These results suggest that the Meta-MFDL predictor has a better generalized performance. In addition, MetaGUN used 261 complete genomes (229 bacteria and 32 archaea) to train the predictor, while our Meta-MFDL just used 120 complete genomes (109 bacteria and 11 archaea) to train the predictor. If we use the same complete genomes as MetaGUN to train Meta-MFDL, Meta-MFDL can achieve better generalized performance than that of MetaGUN.

### 3.2. Effects of Training Data Size and Randomly Sampling

In general, if we use more ORF fragments to train the deep learning model, we can obtain higher prediction accuracy, but it will spend more computational time. In order to investigate the effects of the training data size, we randomly sample different number fragments (e.g., 5000, 10,000, 50,000, 80,000, 100,000, 150,000, and 200,000) from ^Tra^Data700 to build the training dataset in which the number of coding ORF fragments is the same as the noncoding fragments and randomly sample the same number fragments as the training dataset to test the predictors. The results of different training data size are listed in Table S5 available in Supplementary Material. From Table S5, we can see that the metrics of TPR, PPV, ACC, and* F*1 score gradually increase with the increase of size of training data, while the running time significantly increases. For example, the accuracy and running time of Meta-MFDL on the 100,000 and 150,000 fragment training datasets are 92.6%, 92.7%, 1.027 h, and 1.45 h, respectively. The accuracy on the 100,000 dataset just increases 0.1% than 150,000 dataset, while the running time increases 0.423 h. By making a trade-off between the predict metrics and running time, we randomly select 100,000 fragments (i.e., 50,000 coding ORF fragments and 50,000 noncoding ORF fragments) to train our Meta-MFDL predictor in this paper.

In order to investigate the effects of randomly sampling strategy, we randomly sample 50,000 coding ORF fragments and 50,000 noncoding ORF fragments from ^Tra^Data700 ten times and use the 10 CV test to assess the performance of Meta-MFDL.  Table 4 gives the results of ten randomly samplings, in which we can see that randomly sampling strategy has little effect to Meta-MFDL predictor. Thus, the strategy of randomly sampling 50,000 coding ORF fragments and 50,000 noncoding ORF fragments to train the Meta-MFDL model is reasonable and feasible, which can reduce the computational time and gives better prediction results.

### 3.3. Comparison with Individual Feature Classifier

To further verify the effectiveness of Meta-MFDL predictor, we compared it with four other individual feature deep learning classifiers based on the ORF coverage (ORFC), monocodon usage (MC), monoamino acid usage (MA), and Z-curve parameter (ZC) feature descriptors, respectively. The results on the Set700 dataset in 10 CV test are shown in Table 5, from which we can see that the TPR of Meta-MFDL is 3.83, 3.5, 2.94, and 2.52% higher than that of ORFC-DL, MC-DL, MA-DL, and ZC-DL classifiers, respectively; the PPV is little higher than that of ORFC-DL, MC-DL, and MA-DL, and slightly lower than that of ZC-DL;* F*1 score of Meta-MFDL is also bigger than that of other four individual feature classifiers, suggesting that our feature fusion strategy is effective for identifying the gene fragments. The MA and ZC feature descriptors are more powerful than ORFC and MC feature descriptors, meaning that MA and ZC features contribute the most to the overall performance of Meta-MFDL predictor. These results show that Meta-MFDL predictor is effective and robust for predicting metagenomic gene compared with the individual feature DL classifier.

## 4. Conclusions

Identification of genes directly from metagenomic fragments is an important task in annotating metagenomes. However, due to the incomplete and fragmented nature of the metagenomic sequencing data, it is more complicated in metagenomes than in isolated genomes, and the assembly of metagenomes is often not available. Therefore, it is important to develop the computational methods for identifying the coding ORFs from the metagenomics short reads. In this study, based on the DNA sequences, we introduced four kinds of feature descriptors (i.e., ORFC, MC, MA, and ZC) and fused them forming a vector to represent the ORF fragments. Instead of the shallow learning models (e.g., SVM, HMM), we used DSN deep learning architecture model to design the Meta-MFDL predictor for identifying the metagenomic gene fragments. Comparing with the existing Orphelia, FragGeneScan, MGC, and MetaGUN predictors for identifying metagenomics genes on the training datasets and other 13 species independent testing datasets, Meta-MFDL predictor shows strong robust and powerful ability for identifying metagenomic gene fragments, and it represents an intriguing and promising avenue for predicting metagenomic genes. Meta-MFDL software package is available at http://180.208.58.19/Meta-MFDL/.

## Supplementary Material

Table S1. Genomes information in training dataset.
Table S2. Genomes information in testing dataset.
Table S3. TPR, PPV and F1 score of Meta-MFDL, Meta-MFSVM, Orphelia, FragGeneScan, MGC and MetaGUN on the ^Tes^Data700 independent testing dataset.
Table S4. TPR, PPV and F1 score of Meta-MFDL, Meta-MFSVM, Orphelia, FragGeneScan, MGC and MetaGUN on the ^Tes^Data120 independent testing dataset.
Table S5. Results of Meta-MFDL with different training data size.

## Figures and Tables

**Table 1:** The performance of Orphelia, FragGeneScan, Meta-MFSVM, and Meta-MFDL on the Set700 and Set120 training datasets in 10 CV test.

Predictors	Set700			Set120		
	TPR (%)	PPV (%)	*F*1	TPR (%)	PPV (%)	*F*1
Orphelia	88.61 ± 1.89	89.05 ± 1.51	0.888 ± 0.016	83.19 ± 0.98	82.44 ± 1.20	0.847 ± 0.985
Meta-MFSVM	89.59 ± 2.39	90.23 ± 1.34	0.908 ± 0.018	84.70 ± 0.66	85.07 ± 1.26	0.849 ± 0.543
FragGeneScan	90.38 ± 2.23	91.89 ± 1.98	0.918 ± 0.013	86.56 ± 0.71	86.78 ± 0.54	0.868 ± 0.013
Meta-MGPDL	91.47 ± 1.37	93.26 ± 1.97	0.923 ± 0.008	89.28 ± 0.63	90.58 ± 0.61	0.899 ± 0.006

**Table 2:** The overall accuracy (%) of Orphelia, FragGeneScan, MGC, MetaGUN, Meta-MFSVM, and Meta-MFDL on the ^Tes^Data700 independent testing dataset.

Species	Orphelia	Meta-MFSVM	MGC	FragGeneScan	MetaGUN	Meta-MFDL
*A. fulgidus*	89.49	92.25	92.47	93.14	94.30	94.70
*N. pharaonis*	84.73	89.87	89.90	91.67	94.40	94.12
*M. jannaschii*	86.13	91.68	91.69	95.91	95.03	94.65
*B. aphidicola*	94.16	93.43	93.75	95.60	96.24	96.05
*B. pseudomallei*	88.19	90.71	90.76	91.53	94.13	94.15
*B. subtilis*	91.56	92.55	91.78	92.49	93.86	93.89
*C. jeikeium*	86.03	90.15	90.85	91.32	91.31	91.62
*C. tepidum*	83.59	88.48	88.43	88.07	90.68	90.66
*E. coli*	91.50	91.98	92.06	93.45	93.58	93.66
*H. pylori*	92.88	93.30	93.48	94.98	93.81	94.49
*P. aeruginosa*	85.05	92.42	92.47	94.42	94.03	93.89
*P. marinus*	89.16	91.24	91.36	90.82	93.73	93.88
*W. endosymbiont*	78.66	84.70	84.78	75.67	83.69	87.34

*Average*	87.78	90.98	91.06	91.47	92.98	93.31

**Table 3:** The overall accuracy (%) of Orphelia, FragGeneScan, MGC, MetaGUN, Meta-MFSVM, and Meta-MFDL on the ^Tes^Data120 independent testing dataset.

Species	Orphelia	Meta-MFSVM	MGC	FragGeneScan	MetaGUN	Meta-MFDL
*A. fulgidus*	84.05	85.22	85.67	85.92	86.49	87.38
*N. pharaonis*	82.43	82.79	82.96	82.94	83.12	83.03
*M. jannaschii*	82.11	84.25	84.69	84.80	85.23	85.59
*B. aphidicola*	85.91	87.50	88.21	88.96	89.96	90.06
*B. pseudomallei*	84.28	84.73	84.93	85.10	85.37	85.60
*B. subtilis*	88.13	88.36	88.46	88.51	88.60	87.85
*C. jeikeium*	80.95	83.19	84.64	85.96	87.38	91.04
*C. tepidum*	79.17	79.67	80.02	80.16	80.52	79.22
*E. coli*	85.98	86.31	86.56	86.70	86.89	86.91
*H. pylori*	88.09	89.37	90.66	91.42	92.51	92.81
*P. aeruginosa*	83.71	84.28	84.57	84.84	85.15	84.67
*P. marinus*	88.26	88.76	88.98	89.11	89.71	88.73
*W. endosymbiont*	74.75	76.50	77.72	78.51	79.23	79.55

*Average*	83.68	84.69	85.24	85.61	86.17	86.34

**Table 4:** The effects of randomly sampling strategy to the Meta-MFDL predictor in 10 CV test.

Sampling times	TPR (%)	PPV (%)	*F*1
(1)	91.27 ± 1.00	91.79 ± 1.10	0.915 ± 0.009
(2)	91.38 ± 0.92	91.38 ± 0.92	0.916 ± 0.007
(3)	91.64 ± 0.66	91.74 ± 0.88	0.916 ± 0.007
(4)	92.09 ± 0.95	91.73 ± 0.92	0.919 ± 0.008
(5)	92.16 ± 0.77	91.93 ± 0.51	0.920 ± 0.006
(6)	92.22 ± 0.63	91.98 ± 0.53	0.922 ± 0.004
(7)	92.14 ± 0.82	92.57 ± 0.91	0.924 ± 0.006
(8)	92.40 ± 1.26	92.64 ± 0.87	0.925 ± 0.007
(9)	91.92 ± 0.67	93.28 ± 1.50	0.926 ± 0.087
(10)	91.47 ± 1.37	93.26 ± 1.97	0.923 ± 0.008

Average	91.87 ± 0.90	92.23 ± 1.01	0.921 ± 0.015

**Table 5:** Results of Meta-MFDL and four individual feature deep learning classifiers on Set700 dataset in 10 CV test.

Features	TPR (%)	PPV (%)	*F*1
ORFC-DL	87.64 ± 1.24	92.28 ± 1.35	0.899 ± 0.003
MC-DL	87.97 ± 0.75	92.37 ± 1.05	0.899 ± 0.003
MA-DL	88.53 ± 0.49	92.68 ± 0.86	0.905 ± 0.004
ZC-DL	88.95 ± 0.36	93.85 ± 0.75	0.913 ± 0.002
Meta-MFDL	91.47 ± 1.37	93.26 ± 1.97	0.923 ± 0.008
